# Prevalence of gastrointestinal parasites in cattle in Kalasin Province, Thailand

**DOI:** 10.14202/vetworld.2021.2091-2096

**Published:** 2021-08-13

**Authors:** Sirikanda Thanasuwan, Supawadee Piratae, Anupong Tankrathok

**Affiliations:** 1 Department of Veterinary Technology, Faculty of Agricultural Technology, Kalasin University, Kalasin, Thailand; 2 One Health Research Unit, Faculty of Veterinary Sciences, Mahasarakham University, Maha Sarakham, Thailand; 3 Department of Biotechnology, Faculty of Agricultural Technology, Kalasin University, Kalasin, Thailand

**Keywords:** cattle, gastrointestinal parasites, prevalence

## Abstract

**Background and Aim:**

Parasitic infections are one of the major problems to the production of cattle in Thailand. The study was conducted to determine the prevalence of gastrointestinal (GI) parasites of cattle in Kalasin Province, Thailand.

**Materials and Methods:**

A total of 333 fecal samples of cattle were collected directly from the rectum. The fecal samples were subjected to formalin-ethyl acetate concentration methods for examination. The eggs or oocysts were identified based on the morphology and size of the eggs or oocysts.

**Results:**

Out of 333 fecal samples examined, 320 were found positive for GI parasitic infections with a prevalence of 96.09%. Overall, among the prevalence of nematodes, trematodes, and protozoa, the most prevalent parasites were Strongyle-type 278 (84.24%), followed by *Strongyloides* spp. 54 (16.36%) and *Trichuris* spp. 75 (22.73%), while Protozoan oocyst recorded *Eimeria* spp. amounted to 131 (39.7%). *Fasciola* spp. and *Paramphistomum* spp. were 67 (20.30%) and 81 (24.55%), respectively. Most of the positive fecal samples were infected with the double infection which has the highest prevalence rate of about 40.24%, followed by single, three, and 4-5 types of parasites 30.63, 16.82, and 7.21%, respectively.

**Conclusion:**

This study suggests that Kalasin Province is highly endemic for GI parasites and this area may be an important source for an outbreak. Therefore, every household should deworm its cattle and eliminate and control snails as intermediate hosts. Findings from this study provide information that will assist in improving the cattle in Kalasin Province for better production and higher profitability.

## Introduction

The consumption of beef in Thailand amounts to approximately 170,000 tonnes/year. High quality beef from Australia and New Zealand is imported [1]. In addition, the import of frozen beef from the European Union is high, up to 1570 tonnes in the past 7 years [2]. Thailand has seen a sharp increase in the demand for beef. During 2015-2017, there was a high rate of raising crossbred cattle, amounting to 27.15% and beef production increased by 7.48%. For this reason, beef cattle are important animals for the economic development of Thailand. Popularity and demand for domestic cattle consumption tend to increase [1].

Raising beef cattle is considered of great economic importance in Kalasin Province, but the number of beef cattle is not as high as in other provinces. The main reason for this is the geography of Kalasin Province, where most of the province is either used as water reservoirs, dams, or agricultural land. Therefore, there are risk factors for fluke infection [3,4], and there have actually been fluke infections in Kalasin. *Paramphistomum* spp. 38% and *Fasciola* spp. 15%, and other gastrointestinal (GI) parasitic infections were relatively high [5], which may affect the quality and value of beef cattle. In addition, according to the preliminary survey, more than 77.78% of beef cattle in Kalasin Province were not dewormed [2].

Parasitic infections are one of the major constraints to the production of ruminants in tropical and subtropical countries including Thailand [5]. GI parasites inhabit the digestive tract of cattle, which leads to considerable economic loss as a consequence of inappetence, anemia, diarrhea, poor growth, reduced weight gain, impaired reproductive performance, condemnation of affected organs, and mortality in infected animals [6,7]. GI parasitism in cattle is caused by protozoa and helminths. *Eimeria* spp. is a protozoan belonging to phylum Apicomplexa, family of Eimeriidae, which may cause coccidiosis in cattle. This is more often found in young animals. Subclinical or chronic infections can lead to economic losses, diarrhea, or dysentery due to the occurrence of high morbidity and mortality in the young animal, as well as increased costs for prevention and treatment [8,9]. *Trichuris* spp. (also known as whipworm) is a nematodes parasite that attaches to the mucosa of the cecum and colon. *Trichuris* spp. is rarely of clinical significance in ruminants, but it may cause diarrhea, lethargy, ill thrift, weakness, and eventually death [10]. Besides, *Strongyloides* spp. (also known as threadworm) is one of the nematodes within the Rhabditoidea superfamily, their normal shape is small, slender, and live in the small intestine or caeca in some species. *Strongyloides* spp. are able to infect multiple host species worldwide. Normally, there is a specific host for each *Strongyloides* spp. For example, *Strongyloides papillosus* is found in cattle and small ruminants. The calves died within a few minutes of collapsing without any significant preceding clinical signs showing that an animal was suffering from a severe infection with parasites [11]. *Fasciola* spp. is a trematode parasite impacting cattle and cause zoonosis to humans which can lead to alarming health issues. Liver fluke can cause severely acute and sub-acute infections and occurs 2-3 weeks after post-infection which can cause anorexia, yellow conjunctiva, pale, abdominal pain, weight loss, or sudden death [12]. Moreover, *Paramphistomum* spp. is a trematode belonging to the Paramphistomoidea superfamily that parasitizes domestic and wild ruminants, which causes Paramphistomosis. Many species in this superfamily have a different morphology and snails are intermediate hosts [13].

Normally, GI parasitism in ruminants is present throughout the year, with a higher prevalence rate during the rainy season [7]. The climatic conditions such as temperature and humidity are primary factors related to the growth, development, and survival of various parasites, including eggs, larvae, cysts, and oocysts or their intermediate hosts. The estimated mortality rate is about 10% or may exceed 40%, particularly in young animals, while weight loss of 6-12 kg/year per animal may occur [6,14]. In Thailand, the previous studies revealed that GI parasitic infections have a high incidence of parasitism of cattle in the country [6,15-17]. Recently, there has been documentation of the infection of GI among slaughtered cattle that show a high prevalence to 93% of infected cattle with various types of GI parasitism in Mahasarakham Province, geographically adjacent to Kalasin Province [17]. For Kalasin Province, the infection rate of GI parasites of cattle has not been updated for more than a decade. Thus, it is necessary to conduct research to find the infection rate of GI parasites of cattle in this area, which could be important data for evaluation.

Therefore, the study was conducted to determine the prevalence of GI parasite outbreaks in cattle to prevent and control parasites. Adding value and quality in beef cattle production and raising standards to further support domestic consumption and exports.

## Materials and Methods

### Ethical approval

This study was approved by the Institutional Ethical Committee of Kalasin University, Thailand. Samples were collected without any harm to the cattle and were carried out in accordance with standard procedures.

### Study period and location

**Figure-1:**
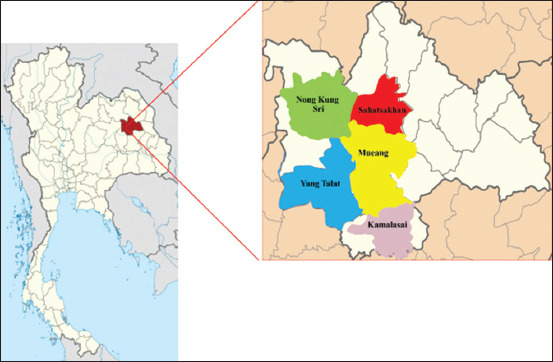
The map of Kalasin Province, Thailand (left), and five districts of sampling sites for cattle feces (right). (Source: https://en.wikipedia.org/wiki/Kalasin_Province).

The study was conducted from August to November 2020 in the district of Mueang, Yang Talat, Kamalasai, Sahatsakhan, and Nong Kung Sri in Kalasin province (Figure-1), Thailand. Kalasin Province occupies an area of 6,947 km^2^ and is located at 16° 26’ 3” and 103° 30’ 33”. The study area was situated at an altitude of about 147 m above mean sea level, where the average temperature was 26.8°C with 1407 mm. of annual rainfall.

### Study design and sampling

Fecal samples of cattle were collected randomly from different locations in Kalasin. Populations of cattle were calculated by a mathematical formula [18], with an expected incidence of 75%, and a confidence level of 95% with 5% of maximum error. The required sample size was calculated to be 288 cattle samples.

### Collection of fecal samples

The study was conducted on 333 cattle samples in five districts of Kalasin Province. Fresh fecal samples were collected directly from the rectum by hand using plastic gloves. The individual fecal samples were brought to the laboratory and processed in the Technology Veterinary Laboratory of Kalasin University. The fecal examination was done by 10% formalin ethylacetate centrifugation [19]. The prevalence was determined using the following equation:


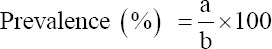


Where, “a” = Number of individuals having a disease at a particular time; “b” = Number of individuals in the population currently at risk [20].

## Results

**Figure-2:**
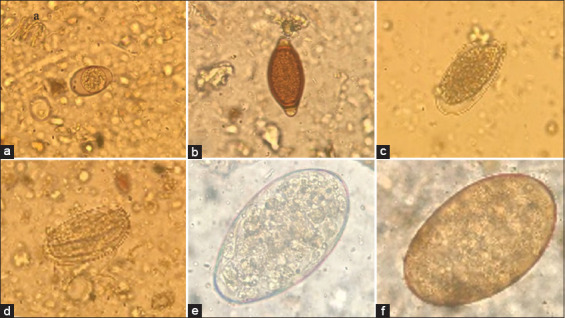
Gastrointestinal parasite presents in beef cattle samples. (a) *Eimeria* spp.; (b) *Trichuris* spp.; (c) Strongyle-type; (d) *Strongyloides* spp.; (e) *Paramphistomum* spp; (f) *Fasciola* spp.

For this study, we collected 333 bovine fecal samples from five districts of Kalasin Province from August to November 2020. GI parasites of cattle were found in almost all areas. The overall prevalence amounted to 96%, and the infection rate was found to be the highest in Sahatsakhan district (100%) (Table-1). The six different parasite species consisted of 3 nematodes, 2 trematodes, and 1 protozoan were detected with the Strongyle-type (84.24%) and were the most prevalent GI parasites overall, followed in frequency by *Paramphistomum* spp. (24.55%), *Trichuris* spp. (22.73%), and *Fasciola* spp. (20.30%). *Strongyloides* spp. (16.36%) was the least prevalent. Besides, protozoan cysts were also found, including *Eimeria* spp. (39.70%) (Table-2 and Figure-2). The infection of Strongyle-type was found to have the highest prevalence in all districts. *Paramphistomum* spp. had the highest prevalence in both Sahatsakhan and Nong Kung Sri (67-68%). *Fasciola* spp. (51.22%) and *Eimeria* spp. (50.70%) had the highest prevalence in *Sahatsakhan* and Yang Talat. Finally, *Trichuris* spp. (33.33%) and *Strongyloides* spp. (25.0%) had the highest prevalence in Kamalasai district (Table-2). A total of 1-4 different types of parasites were detected. The most common coinfection was two types of parasite (40.24%), followed by 1 (30.63%), 3 (16.82%), and >4 (7.21%) types, respectively (Table-3).

**Table-1:** Prevalence of GI infection by genera in beef cattle in Kalasin Province, Thailand (five districts).

District	Number of animals	Number of positive	Prevalence (%)
Mueang	139	136	97.84
Sahatsakhan	43	43	100
Yang Talat	71	66	92.96
Nong Kung Sri	41	39	95.12
Kamalasai	39	36	92.30
Total	333	320	96.09

GI=gastrointestinal

**Table-2:** Prevalence (%) of GI infection among beef cattle in Kalasin, Thailand.

Parasites	Prevalence (n=330)	District				

		Mueang (n=139)	Sahatsa-Khan (n=43)	Yang Talat (n=71)	Nong Kung Sri (n=41)	Kamalasai (n=36)
Nematodes						
Strongyle-type	278 (84.24)	115 (82.73)	29 (67.44)	54 (76.05)	14 (34.14)	26 (72.22)
*Strongyloides* spp.	54 (16.36)	19 (13.66)	10 (23.26)	8 (11.27)	8 (19.51)	9 (25.0)
*Trichuris* spp.	75 (22.73)	30 (21.58)	10 (23.26)	19 (26.76)	4 (9.76)	12 (33.33)
Trematodes						
*Fasciola* spp.	67 (20.30)	33 (23.74)	13 (30.23)	0 (0.0)	21 (51.22)	0 (0.0)
*Paramphistomum* spp.	81 (24.55)	3 (2.16)	29 (67.44)	13 (18.31)	28 (68.29)	8 (22.22)
Protozoan						
*Eimeria* spp.	131 (39.70)	54 (38.84)	22 (51.16)	36 (50.70)	6 (14.63)	13 (36.11)

GI=Gastrointestinal

**Table-3:** Prevalence of mixed and coinfection of parasites among beef cattle in Kalasin Province.

Parasites	No. examined	Coinfection with GI parasites (%)			

		Single	2	3	>4
Mueang	139	51 (36.69)	56 (40.29)	23 (16.55)	3 (2.16)
Sahatsakhan	43	9 (20.93)	19 (44.19)	10 (23.26)	5 (11.63)
Yang Talat	71	18 (25.35)	33 (46.48)	7 (21.21)	9 (12.68)
Nong Kung Sri	41	10 (24.39)	17 (41.46)	8 (19.51)	3 (7.32)
Kamalasai	39	14 (35.90)	9 (23.08)	8 (20.51)	4 (10.26)
Total	333	102 (30.63)	134 (40.24)	24 (7.21)	56 (16.82)

GI=Gastrointestinal

## Discussion

GI parasitic infections are a major constraint to cattle production worldwide [21-23] and are more problematic in developing countries, especially those related to climate, nutrition, and poor sanitation, including Thailand [16,17]. The high prevalence of GI infections in cattle in Kalasin is in line with the results in cattle in Mahasarakham (93%) [17], but the data differed from cattle in Udonthani (60%) [16]. The results of this study showed a high prevalence of Nematodes infection, especially Strongyles were the most frequent compared with other genotypically similar parasites [21,23,24]. The strongyles order was highly prevalent. These consist of four superfamilies, Strongyloides, Trichostrongyloidea, Ancylostomatoidea, and Metastrongyloidea, and a total of 29 genera which have many species [25]. In addition, the factors influencing the prevalence of GI parasites are the standards for the management of grazing, anthelmintics as well as economic conditions and the level of education of farmers [26]. *Fasciola* spp. and *Paramphistomum* spp. infections cause severe rumen and liver flukes in cattle farming, including weight loss, reduction of milk production, low fertility rates, and maybe mortality. Rumen and liver flukes are both normal flukes impacting cattle and other livestock in Thailand [27]. Noticeably, researchers have found that the density of flukes is dependent on the population of intermediate snail hosts, and there are two major important intermediate snail hosts of fluke, including *Bulinus* spp. and *Planorbis* spp. [28]. These two intermediate snail hosts play an active role in the transmission of snail-borne trematode infections. In addition, the prevalence rate of flukes is associated with the grazing system, nutrition status, and environment [29]. In this study, Nong Kung Sri district had the highest infection rate with *Fasciola* spp. because the district is in the vicinity of Lampao Dam. Farmers let their cattle graze in the vicinity of the dam area, resulting in relatively high rates of infection with the flukes of *Fasciola* spp. (51%) and *Paramphistomum* spp. (68%). Because such parasites have a high incidence near water sources or dams [3,4,27,30]. Yang Talat District and Kamala Sai had lack infections with *Fasciola* spp. because the sampling area was not in the vicinity of the dam, therefore, there is a possibility of finding a small number of flukes or none at all, or there might be a similar form of parasitic infection. There are two types of flukes, namely, *Paramphistomum* spp. and *Fasciola* spp. Infections with those two flukes will reduce the chance of infection with other types of parasites. This is because the two parasites share the same intermediate host. Therefore, the competition for survival between the flukes may be the reason why *Paramphistomum* spp. was found in Yang Talat District and Kamalasai district, but *Fasciola* spp. was not found. Nong Kung Sri had the lowest infection with strongyle parasite, probably because most farmers tended to raise cattle freely in pens or with better pen management than in other districts, which causes less strongyles infection than in other districts. The same result was found in Kaewnoi. In bulls, the main infection was caused by *Paramphistomum* spp. (97.17%), but strongyles infection was low (26.29%) [19].

*Fasciola* spp. are associated with diseases in cattle, sheep, and goats. However, this study found a low prevalence of *Fasciola* spp. infections, fasciolosis has been identified by the WHO as a remerging neglected tropical disease associated with endemic and epidemic outbreaks of diseases in human populations [19]. Our findings indicate that the high prevalence of *Fasciola* spp in Kalasin Province may relate to the location of Kalasin Province which is adjacent to Lampao dam, resulting in the appropriate temperature and humidity for promoting the intermediate host population growth such as intermediate snail hosts and water vegetables [31,32]. Fascioliasis is a zoonosis and has a high prevalence among herding communities in developed countries because of their close contact with livestock animals [31]. Besides, we found high infection rates in cattle infected with *Paramphistomum* spp. in Nong Kung Sri and Sahatsakhan (67-68%). Adult worms of paramphistome are generally considered subclinical in the host and thousands of adult worms may not stimulate clinical signs [33] but the migration of larvae, which live in the duodenal mucosa intestine, cause severe enteritis, possible necrosis, hemorrhage, anoxia, polydipsia, and mortality [34]. Moreover, the mortality due to migration of larvae is very high and sometimes reaches 80-90% in livestock animals [35].

This study also detected the protozoa *Eimeria* spp. (39%), which is higher than in all prior reports (3-21%) [15,16]. The reason for the high prevalence rate of protozoa is that the stalls of the cattle are overcrowded, adult and calf stay together, lack of good farm management, and sanitation as well as cattle have not been dewormed. Coccidiosis is the most pathogenic intestinal disease that causes high morbidity in domestic animals, by *Eimeria* spp. [36]. This protozoan is infesting cattle and other ruminants worldwide. It can invade the small and large intestine of hosts causing anemia, electrolyte losses, and diarrhea [37]. Adult animals normally show subclinical signs but can be a reservoir for younger animals. The calf can suffer from a range of symptoms, including weight loss, reduced weight gain, apathy, diarrhea, dysentery, dehydration, debilitation, and death [38]. *Eimeria* spp. infection was only seen in young animals because adult animals, after having been infected *Eimeria* spp., their immune system was stimulated to protect themself [39]. However, the immune system of the animal was suppressed due to several factors such as stress from the overcrowding of the stalls or animal transport, poor nutrition, or intercurrent diseases, or they are subject to heavy infection that might cause clinical disease [8,40]. In addition, these protozoa can adapt to different climatic conditions, environments and they have a long life within oocysts. In addition, oocysts can contaminate pastures and water that protozoa will develop in to sporulate oocyst. Finally, adult protozoans developed in cattle [40].

Moreover, this study showed two types of mixed parasites infection that reach 40.24% of the animals and the rest presented infections by single (30.63%), three (16.82%), or more parasites (7.21%), respectively. Due to the high prevalence rate, we selected a sample that has never been dewormed for more than 1-year [16]. However, almost all farmers do not have their own grass or cut grass near the canal, or cattle were released for feed and take water near the dam area. Based on inquiries, each area did not deworm its cattle because the cattle had to be moved a considerable distance. Some farmers have large numbers of cattle that are not easy to move. To control GI parasite infection in cattle requires a comprehensive knowledge of the disease epidemiology and an understanding of pasture management, farm management practices, and agroclimatic conditions such as temperature and humidity [41].

## Conclusion

GI parasites are detected in high prevalence among cattle in Kalasin Province, Thailand with commonly occurs in mixed infections. The presence of *Fasciola* spp. shows a risk factor to the health public. The dominant type of GI parasites is strongyle order. Therefore, this study indicated that the cattle in Kalasin Province have been infected with GI parasites at a very high rate. This area could be an important source of parasites causing an outbreak in the future. To avoid infections by parasites, the cattle should be dewormed every 6 months, the pens should be managed properly and the area where cattle are kept should not be wet.

## Authors’ Contribution

ST: Planned and designed the experiment; analysis of data drafted and revised the manuscript. SP: Participated in manuscript drafting and analyzing data. AT: Analyzed data and participated in the fieldwork. All the authors have read and approved the final manuscript.

## References

[1] Bunmee T, Chaiwang N, Kaewkot C, Jaturasith S (2018). Current situation and future prospects for beef production in Thailand-a review. Asian-Australas J. Anim. Sci.

[2] Phongchongmit T, Norrapoke T (2019). The study on situation of beef cattle and satisfaction of farmers in Kalasin province. KKU Vet J.

[3] Thanasuwan S, Piratae S (2019). Outbreak of *Fasciola* spp. causing zoonotic diseases. Khon Kaen Agric. J.

[4] Jaja I.F, Mushonga B, Greenc E, Muchenje V (2017). Seasonal prevalence, body condition score and risk factors of bovine fasciolosis in South Africa. Vet. Anim. Sci.

[5] Aunpromma S, Papirom P (2006). Survey of internal parasites of adult native cattle from Amphur Sahasakhan Kalasin province. KKU. Vet. J.

[6] Jittapalapong S, Sangwaranond A, Nimsuphan B, Inpankaew T, Phasuk C, Pinyopanuwat N, Chimnoi W, Kengradomkit C, Arunwipat P, Aewith T (2011). Prevalence of gastrointestinal parasites of dairy cows in Thailand. Kasetsart J.

[7] Marskole P, Verma Y, Dixit A.K, Swamy M (2016). Prevalence and burden of gastrointestinal parasites in cattle and buffaloes in Jabalpur, India. Vet. World.

[8] León J.C.P, Delgado N.U, Florez A.A (2019). Prevalence of gastrointestinal parasites in cattle and sheep in three municipalities in the Colombian Northeastern Mountain. Vet. World.

[9] Volpato A, Tonin A.A, Machado G, Stefani L.M, Campigotto G, Glombowsky P, Galli G.M, Favero J.F, Silva A.S (2017). Gastrointestinal protozoa in dairy calves:Identification of risk factors for infection. Rev. MVZ Córdoba.

[10] Wideman G.N (2004). Fatal *Trichuris* spp. infection in a Holstein heifer *Trichuris* spp. infection in a Holstein heifer *Trichuris* persistently infected with bovine viral diarrhea virus. Can. Vet. J.

[11] Thamsborg S.M, Ketzis J, Horii Y, Matthews J.B (2017). *Strongyloides* spp. infections of veterinary importance. Parasitology.

[12] Bennett R, Ijpelaar J (2005). Updated estimates of the costs associated with thirty four endemic livestock diseases in Great Britain:A note. J. Agric. Econ.

[13] El-Bahy N.M, Bazh E.K, Abdel Azizn A.R, Elkhtam A (2017). New approach to molecular characterization of *Paramphistomum cervi* and *Carmyerius gregarious* and comparative analyses with selected trematodes. Parasitol. Res.

[14] Chavhan P.B, Khan L.A, Raut P.A, Maske D.K, Rahman S, Podchalwar K.S, Siddiqui M.F.M (2008). Prevalence of Nematode parasites of ruminants at Nagpur. Vet. World.

[15] Wongsawang W, Sanyutitham S, Nakthong C (2014). The survey of gastrointestinal parasites in beef, Sai-Yok district, Kanchanaburi province. Appl. Anim. Sci.

[16] Yuwajita C, Pruangka S, Sukwong T (2014). Prevalence of gastrointestinal parasites of cattle in Udon Thani, Thailand. Khon Kaen Agric. J.

[17] Sakwiwatkul K, Chaikong C, Thamwan C, Wattanakham P, Kessimlee P, Kuphukhiaw P (2017). Prevalence and risk factor of infection internal parasites found in cattle from slaughterhouse in Mahasarakham province, Thailand. Khon Kaen Agric. J.

[18] Mpofu T.J, Nephawe K.A, Mtile B (2020). Prevalence of gastrointestinal parasites in communal goats from different agro-ecological zones of South Africa. Vet. World.

[19] Kaewnoi D, Wiriyaprom R, Indoung S, Ngasam R (2020). Gastrointestinal parasite infections in fighting bulls in South Thailand. Vet. World.

[20] Thrusfield M (2005). Veterinary Epidemiology.

[21] Hamid P.H, Kristianingrum Y.P, Prastowo J, Silva L.M.R (2016). Gastrointestinal parasites of cattle in Central Java. Am. J. Anim. Vet. Sci.

[22] Chowdhury R, Sen A, Kar J, Nath S.K (2017). Prevalence of gastrointestinal parasitism of cattle at Chandaniash Upazilla, Chittagong, Bangladesh. Int. J. Adv. Res. Biol. Sci.

[23] Obi C.F, Akata M.C, Ezubelu O.J (2020). Prevalence of gastrointestinal helminth parasites of trade cattle in Aguata and Orumba South Local Government Areas, Southeastern Nigeria. J. Parasit. Dis.

[24] Murthy C.M.K, Souza P.E.D (2016). Prevalence of gastrointestinal parasites in bovines in Bangalore district, Karnataka. J. Parasit. Dis.

[25] Bowman D.D (2009). Georgis'Parasitology for Veterinarians.

[26] Baihaqi Z.A, Widiyono I, Nurcahyo W (2019). Prevalence of gastrointestinal worms in Wonosobo and thin-tailed sheep on the slope of Mount Sumbing, Central Java, Indonesia. Vet. World.

[27] Japa O, Siriwechviriya P, Prakhammin K (2020). Occurrence of fluke infection in beef cattle around Phayao Lake, Phayao, Thailand. Vet. World.

[28] Hamba L.M, Ayuni R, Vanda H, Amiruddin A, Athaillah F (2019). Occurrence of *Fasciola gigantica* and *Paramphistomum* spp. infection in Aceh cattle. E3S Web of Conferences No. 151, 1^st^ ICVAES.

[29] Shinggu P.A, Olufemi O.T, Nwuku J.A, Baba-Onoja E.B.T, Iyawa P.D (2019). Liver flukes egg infection and associated risk factors in white fulani cattle slaughtered in Wukari, Southern Taraba State, Nigeria. Adv. Prev. Med..

[30] Goiyram N, Pimpukdee K, Saksangawong C (2016). The comparison of liver fluke prevalence in native cattle between Thai-Laos and Thai-Cambodia border. J. Sci. Technol. MSU.

[31] Nyindo M, Lukambagire A.H (2015). Fascioliasis:An ongoing zoonotic trematode infection. Biomed. Res. Int.

[32] Rinca K.F, Prastowo J, Widodo D.P, Nugrahe Y.R (2019). Trematodiasis occurrence in cattle along The Progo river, Yogyakarta, Indonesia. Vet. World.

[33] Nurhidayah N, Satrija F, Retnani E.B, Astuti D.A, Murtin S (2020). Prevalence and risk factors of trematode infection in swamp buffaloes reared under different agro-climatic conditions in Java Island of Indonesia. Vet. World.

[34] Khedri J, Radfar M.H, Borji H, Mirzaei M (2015). Prevalence and intensity of *Paramphistomum* spp. in cattle from South-Eastern Iran. Iran J. Parasitol.

[35] Maitra A, Yadav C.L, Sanjukta R.K (2014). Seasonal prevalence of paramphistomosis in domestic ruminants in different agro-climatic zones of Uttarakhand, India. Asian Pac. J. Trop. Dis.

[36] Das M, Deka D.K, Sarmah P.C, Islam S, Sarma S (2015). Diversity of *Eimeria* spp. in dairy cattle of Guwahati, Assam, India. Vet. World.

[37] Hassan N.M.F, Farag T.K, Abu El Ezz N.M.T, Abou-Zeina H.A.A (2019). Prevalence assessment of gastrointestinal parasitic infections among goats in Giza Governorate, Egypt. Bull. Natl. Res. Cent.

[38] Morgoglione M.E, Bosco A, Maurelli M.P, Alves L.C, Saralli G, Bruni G, Cringoli G, Rinaldi L (2020). A 10-year surveillance of *Eimeria* spp. in cattle and buffaloes in a Mediterranean area. Front. Vet. Sci.

[39] Alcala-Canto Y, Figueroa-Castillo J.A, Ibarra-Velarde F, Vera-Montenegro Y, Valencia M.E.C, Alberti-Navarro A (2020). First database of the spatial distribution of *Eimeria* species of cattle, sheep and goats in Mexico. Parasitol. Res.

[40] Ola-Fadunsin S.D, Rabiu M, Hussain K, Sanda I.M, Ganiyu I.A (2020). Epidemiological studies of *Eimeria* species of cattle in Ilorin, North-Central Nigeria. Ann. Parasitol.

[41] Gunathilaka N, Niroshana D, Amarasinghe D, Udayanga L (2018). Prevalence of gastrointestinal parasitic infections and assessment of deworming program among cattle and buffaloes in Gampaha district, Sri Lanka. Biomed. Res. Int.

